# Differences in anthropometric variables and muscle strength in relation to competitive level in male handball players

**DOI:** 10.1371/journal.pone.0261141

**Published:** 2021-12-09

**Authors:** Marcin Lijewski, Anna Burdukiewicz, Aleksandra Stachoń, Jadwiga Pietraszewska

**Affiliations:** Department of Biological and Medical Foundations of Sport, University School of Physical Education in Wrocław, Wrocław, Poland; Universita degli Studi di Milano, ITALY

## Abstract

Somatic characteristics manifested in different body morphology have great importance for the selection of athletes in most sports. The aim of our study is to evaluate the differences in anthropometric variables and isometric strength of handball players presenting different levels of sports competence, and to study the discriminative power of selected morphological characteristics that do not change in the training process. The study included the results of anthropometric measurements routinely used to monitor athletes, and body proportion indices were calculated. Fat percentage was assessed using the BIA, whereas body build was assessed using the Heath-Carter method. Measurements of right and left hand grip strength and back strength were taken. The results of measurements and calculations were analyzed using statistical methods. It was shown that players presenting the highest level dominate by the overall size and massiveness of the body, characteristics ensuring an advantage in direct confrontation. The size of subcutaneous fat tissue and percentage of body fat varied poorly between athletes in each group. Muscle strength assessed under static conditions shows a gradient in magnitude across teams from higher to lower rank, but the differences are not statistically significant. The same somatotype (balanced mesomorph) was present in all groups. Athletes presenting high sports level are characterized by body proportions that determine biomechanical conditions conducive to optimizing the structure of movements important in handball. Stepwise discriminant analysis showed that throwing-related characteristics (hand length, upper arm length, upper limb span, lower limb length) account for 88% of the variance in team ranking and can be used to identify the morphological predisposition of adepts to play handball.

## Introduction

Athletes training for different sports vary in fitness and physical performance. Somatic characteristics demonstrate great importance for the selection of adepts in most disciplines, which is reflected in the athlete body morphology [1, 2]. The role that body height and body shape play in increasing the likelihood of success varies from sport to sport. In some sports, we observe the occurrence of individuals with almost extreme body types (e.g. athletic running), while other competitions are represented by an almost somatically homogeneous groups of players (e.g. basketball players).

Handball is a highly dynamic contact game requiring a high level of aerobic and anaerobic fitness, as the game involves numerous actions characterized by high intensity (sprints, jumps, throws and physical confrontations) alternating with periods of low activity (e.g. standing, walking and jogging). Varied motor demands are reflected in the athlete body morphology [3, 4]. Apart from anthropometric features, factors determining an athlete’s success are: the level of strength, power and velocity of throws, as well as technical and tactical skills and mental qualities [5, 6]. Previous studies have argued that a high level of muscle strength is necessary for effective use of technique during handball competition [7, 8]. Hand grip strength plays an important role in catching and throwing a ball [9] and because of that, measurement of this characteristic is assumed to be an objective indicator of the functional integrity of the upper limb [10]. In turn, the strength of trunk muscles determines the appropriate stabilization and coordination of the upper and lower body during throwing, contributing to its effectiveness [11].

Exercise loads create differences between athletes in body composition and functional characteristics [12], that generates interest among practitioners and researchers in the key factors and characteristics to distinguish high- and low-class players. The study of the body morphology in top-level athletes is important to accurately determine the criteria for selecting young people for a sport, because somatic characteristics are shaped by endogenous genetic factors and exogenous environmental factors, which may include training. Body fatness and musculature are highly plastic and show the greatest changes under the influence of exogenous factors (training and diet), while skeletal size shows relatively little change [13, 14].

Many studies have shown that handball players differ from the general population in body size and shape (mean of features, lower variance), so it should be assumed that somatic characteristics are one of predictors of success in this sport [15]. Comparative analyses of morphological characteristics of player groups presenting different levels of sports competence are also a source of similar statements. Because the results of such analyses provide targeted gradients in the magnitude of somatic characteristics, the effect of selection based on body morphology can be inferred from these [16]. The systematic evaluation of anthropometric and functional profiles enables the collection of objective data that can be used to identify talents among young sports adepts [17], yet interpretation of measurement data in younger age groups (juniors) in handball requires caution, as the observed differences may result from developmental maturation. Despite these caveats, it must be concluded that systematic monitoring of changes in morphology and performance promotes maximizing the chance of success and enables the development of structured youth training programs. In addition, understanding the differences that exist between elite and non-elite athletes allows coaches to compare athletes and classify them in terms of desired characteristics.

Studies of men’s handball teams have shown that both physiological and physical characteristics differentiate players from teams presenting league rankings. Research of Norwegian handball players from national team and the 1^st^ division have shown that better performance in tests of throwing velocity, 20-m sprint, countermovement jump, 3000-m running and bench press are achieved by players ranked higher [18]. The method of stepwise discriminant analysis showed that stature and mean power during the Bosco test were the most important characteristics in elite handball players in the first league of the Greek championship, accounting for 54.6% of the variance in team ranking [19]. The analysis of anthropometric profiles and physical performance including heart rate, sprint ability, jumping performance, throwing velocity, and endurance performance in terms of playing position and playing class revealed a close relationship between these variables in players from the German first and second leagues [7]. In addition to that, anthropometric studies of Portuguese handball players representing different levels of performance have shown that morphological optimization contributes significantly to success in handball. The level of performance was related to the size, shape and composition of the body, i.e. the structural characteristics that provide the conditions for effective play in a specific playing position. Application of discriminant analysis showed that upper limb length, arm and forearm circumference, as well as body composition were the factors that contributed to optimal performance the most [4]. Other studies have found that elite athletes have more favorable anthropometric characteristics, performance and higher throwing velocity compared to lower ranked teams [20, 21].

Based on the literature discussed above, it can be concluded that maximizing the chance of success requires developing a comprehensive system for identifying and developing talent, and involves updating the values of variables that characterize elite players in many aspects. One of these variables is body structure, and although it is not the only factor determining sporting success, the size and shape of the players’ bodies are important in handball and differ significantly from the general population, and also shows variation depending on the playing position [12, 22]. The aim of our study is to evaluate the differentiation in somatic characteristics and isometric strength of handball players presenting various levels of sports competence, and to investigate the discriminative power of selected morphological characteristics that do not change in the training process.

## Material and methods

### Participants

The study involved 70 handball players (n = 70; age 23.9 ± 5.80 years), presenting different levels of sport competence, including 20 players from Super League clubs (group 1) (29.8± 6.48 years old, 17.6 ± 6.65 10 ± 3 years of handball experience), 20 players from club teams of 1^st^ League (group 2) (22.9 ± 4.08 years old, 11.6 ± 4.39 years of handball experience) and 30 academic athletes (group 3) (20.2 ± 1.13 yrs old, 9.8 ± 1.26 years of handball experience). The analysis of variance used showed that the athletes in group 1 were significantly older compared to the subjects in groups 2 and 3. Moreover, they were also characterized by significantly longer handball experience. The differences between the other groups (2 and 3) did not reach the threshold of statistical significance.

Measurements were taken at the end of the preparatory period, before the start of the competition season. All participants visited the laboratory once and underwent a series of anthropometric and strength measures. The study was approved by the Ethics Committee of the University School of Physical Education in Wrocław, Poland, and conducted according to the requirements stipulated in the Declaration of Helsinki (2/2020). Participants were fully informed about all experimental procedures and written informed consent was obtained from all of them. Surveys were used to collect information regarding date of birth, length of training experience, supplements used, and presence of injuries.

### Measurements and calculations

All anthropometric measurements were taken in the morning hours. Experienced anthropometrists performed all anthropometric examinations according to measurement protocols established by the International Society for the Advancement of Kinanthropometry (ISAK). Each anthropometrist took the same measurements and was assisted by a recorder. All bilateral measurements were obtained from the right side of the body. Anthropometric measurements were taken using anthropological instruments (anthropometer, sliding caliper—Martin type, spreading caliper, skinfold caliper, plastic tape) by GPM Siber Hegner Machinery Ltd. (Zurich, Switzerland), body weight was assessed using an electronic scales with an accuracy of 0.1 kg (Fawag, Lublin, Poland). Each measure was taken two times by the same investigator. Technical error of measurement was <3% for skinfolds, and <1% for breadths, lengths and girths.

The study included the results of measurements routinely used to monitor athletes [15]. Heights, lengths, widths, and circumferences were measured to the nearest 0.1 cm: body height, arm span, sitting height, lower limb height to *trochanterion* point, upper limb length, arm length, forearm length, hand length, biacromial breadth, biiliocristal breadth, hand breadth between *metacarpale radiale* and *metacarpale ulnare* points, biepicondylar humerus breadth, biepicondylar femur breadth. The following girths were measured: chest at the level of the *mesosternale* point, arm (relaxed and flexed/tensed), forearm, thigh taken 1 cm below the level of the gluteal fold and calf. Skinfold sites were landmarked at the triceps, subscapular, forearm, supraspinale, front thigh and medial calf on the right side of the participant’s body. All sites were then measured using caliper with 10 g×mm^−2^ constant pressure.

The measured characteristics were used to calculate the following quotient indices: body mass index (BMI)—body mass/body height^2^ [kg/m^2^], relative arm span—arm span/body height, cormic index—sitting height/body height, relative lower limb length—lower limb length/body height, relative upper limb—upper limb length/body height, relative arm length—arm length/body height, relative forearm length—forearm length/body height, relative hand length—hand length/body height, brachial index—forearm length/arm length, hand breadth/hand length, relative biacromiale breadth—biacromiale breadth/body height, relative biiliocristale breadth—biiliocristale breadth/ body height,

The ascertained values were used to determine somatotype according to Sheldon’s method as modified by Heath and Carter [23]. Somatotype Calculation and Analysis software classified the average somatotype of each group and illustrated the outcome in a somatotype chart [24].

Measurements of right and left hand grip strength were also taken, that plays an important role when catching and throwing a ball or other equipment in various sports [25]. The grip strength of the hand muscles was measured using a hand grip dynamometer (T.K.K.5001, Takei Scientific Inst. Co., Ltd., Niigata City, Japan). The purpose of this test was to measure the maximum isometric strength of the hand and forearm muscles [26]. The straightened upper limb was lowered downward during the measurement. Movements of the arm or wrist were not allowed. The subject squeezes the dynamometer with maximum isometric effort, which is maintained for about 5 seconds. Peak developed strength in kilograms [kg] was recorded. The back strength was measured by using back and leg dynamometer (T.K.K.5402, Takei Scientific Inst. Co., Ltd., Niigata City, Japan). The subjects stands on the base of the dynamometer and legs and backs were straightened to allow the bar to be at the level of the patella [27]. For both the experiments each volunteer performed 3 trials with a rest of 30 seconds between each trial. The highest score of the trials was recorded in kilograms as the corresponding hand grip and back strength.

Force measurements illustrate the functional capacity of skeletal muscles. To assess the magnitude of absolute strength in relation to the body size of the athletes, relative strength was calculated, which is particularly useful when comparing individuals with different body dimensions. Relative strength was calculated as the sum of the best efforts for each hand and back strength divided by body mass and expressed as kg·kg^-1^ of body mass.

### Statistical analysis

The statistical analysis was performed with the use Statistica 13 package (Dell Inc., Tulsa, Oklahoma, United States). Descriptive statistics were applied to quantitatively analyze the collected data. Shapiro–Wilk test was used to examine the distributions in the analyzed characteristics. Variance analysis and Tukey’s HSD test were used to assess the intergroup differences in body structure and in the analyzed anthropometric and functional characteristics. Data in the text and tables are presented as mean and standard deviation. The significance level was set at p < 0.05. Differences between the somatotypes of the groups were examined using Somatotype Analysis of Variance (SANOVA) [24].

Discriminant function analysis determined which variables are the best predictors of high levels of athletic capabilities in handball players. Anthropometric characteristics that are not modified by training (body height, upper limb span, lower limb length, upper arm length, forearm length, and hand length) were selected for calculation. The paper uses forward stepwise analysis, in which the discrimination model is built step by step.

## Results

In most of the analyzed somatic characteristics, the highest values are found in handball players presenting the highest level and the lowest–in subjects from group 3 (Table 1). Players included in group 2 present intermediate values of the variables discussed. Super League athletes have significantly greater body weight and arm circumferences compared to athletes in group 3. They also have significantly greater upper limb span and longer lower and upper limbs. The torso dimensions (shoulder width and hip width) show a similar direction of difference. A statistically significant difference in arm and hand length occurs when comparing group 1 with the others. Body height and sitting height, similar to hand, elbow, and knee width, as well as forearm, thigh, and lower leg circumferences also assume the highest values in subjects from group 1, but the intergroup differences are statistically insignificant.

**Table 1:** Statistical characteristics and inter-group differences of the morphological traits in handball players (SD–standard deviation).

Group	1		2		3		*p*
Variable	Mean	SD	Mean	SD	Mean	SD	
Anthropometry							
Body mass [kg]	94.0^a^	12.58	89.1	13.21	82.6	8.21	0.004
Body height [cm]	188.3	7.32	185.4	6.61	184.1	6.54	0.120
Arm span [cm]	194.3^a^	9.16	188.3	7.35	185.4	8.63	0.003
Sitting height [cm]	98.1	3.91	97.7	2.87	97.7	3.07	0.888
Lower limb height [cm]	100.4^a^	5.11	97.8	5.27	95.9	5.03	0.017
Upper limb length [cm]	83.88^a^	4.38	80.70	2.97	80.05	4.05	0.004
Arm length [cm]	36.20^a^^b^	1.71	34.42	1.68	34.34	1.67	0.001
Forearm length [cm]	26.98	1.94	26.49	1.25	26.11	1.94	0.262
Hand length [cm]	20.71^a^^b^	1.31	19.79	0.85	19.60	1.06	0.003
Biacromial breadth [cm]	44.4^a^	2.16	43.8	2.69	42.3	2.96	0.026
Biiliocristal breadth [cm]	30.7^a^	2.16	29.9	2.37	28.6	1.65	0.005
Hand breadth [cm]	9.2	0.58	8.9	0.60	8.8	0.58	0.053
Humerus breadth [cm]	7.6	0.58	7.4	0.55	7.2	0.47	0.071
Femur breadth [cm]	10.5	0.53	10.3	0.83	10.2	0.53	0.229
Arm girth relaxed [cm]	33.8^a^	3.47	33.0	2.27	31.2	1.96	0.004
Arm girth flexed/tensed [cm]	37.3^a^	3.43	36.2	2.47	35.0	2.36	0.024
Forearm girth [cm]	29.7	1.88	29.5	1.87	28.9	1.66	0.265
Thigh girth [cm]	62.4	4.30	61.7	5.62	59.7	2.97	0.094
Calf girth [cm]	40.6	3.17	40.7	2.63	39.5	2.55	0.239
Subscapular skinfold [mm]	10.0	2.61	10.7	2.90	9.4	2.28	0.237
Supraspinale skinfold [mm]	10.9	3.83	11.0	5.35	9.6	4.05	0.499
Triceps skinfold [mm]	4.3^a^	1.33	4.2^a^	1.10	6.0	3.10	0.008
Forearm skinfold [mm]	2.8^a^	0.44	3.4	0.76	3.6	1.05	0.009
Thigh skinfold [mm]	8.6	1.70	8.4^a^	1.94	10.1	3.28	0.045
Inch skinfold [mm]	4.8	1.28	4.6^a^	1.44	5.7	2.06	0.047
Strength							
Hand grip strength R [kg]	61.6	9.26	55.5	13.87	53.6	14.79	0.199
Hand grip strength L [kg]	56.0	10.91	52.2	12.30	49.4	13.04	0.231
Back strength [kg]	159.4	16.25	147.9	23.12	146.8	27.73	0.195

^a^ significantly different from 3.

^b^ significantly different from 2.

The thickest skinfolds on the torso are found in 1^st^ League handball players. The least accumulation of subcutaneous fat on the trunk characterizes academic players. The mentioned characteristics do not show statistically significant variation. Skinfolds on limb segments significantly differentiate the study groups. Players presenting the lowest level of athletic competence show the greatest fatness of the arm, forearm, thigh, and lower leg. Subjects in group 1 have the least forearm fatness. The other skinfolds analyzed are the thinnest in handball players in group 2. The magnitude of hand grip strength and back muscles showed no statistically significant differences. The lowest static strength development was found in group 3. Group 1 professionals had the best results in the strength tests.

Table 2 shows the statistical characteristics of handball players’ body proportions by sport level. Professional players presenting the highest level in comparison to the other groups are characterized by significantly greater massiveness of the body assessed by BMI index, greater range of upper limbs and longer lower and upper limbs in relation to body height. Upper limb segment lengths after precipitating the effect of body height are also the greatest in this group. In contrast, the brachial index value is the lowest, indicating the presence of a relatively short forearm in relation to arm length. Hip width in relation to body height is significantly higher in the discussed group. The subjects in group 2 are characterized by the highest values of brachial index and the smallest relative arm length. Athletes in the third group have the greatest sitting height in relation to body height.

**Table 2:** Statistical characteristics and inter-group differences of the morphological traits in handball players (SD–standard deviation).

Group	1		2		3		*p*
Variable	Mean	SD	Mean	SD	Mean	SD	
BMI	26.45^a^	2.77	25.81	2.49	24.37	2.27	0.018
Relative arm span	103.18^a^	2.50	101.58	2.34	100.70	2.64	0.006
Cormic index	52.11^a^	1.28	52.71	1.18	53.07	0.87	0.018
Relative lower limb length	53.31^a^	1.14	52.74^a^	1.57	52.08	1.64	0.025
Relative upper limb	44.53^a^	1.16	43.54	1.08	43.46	1.26	0.007
Relative arm length	19.22^a^^b^	0.51	18.57	0.66	18.65	0.57	0.001
Relative forearm length	14.31	0.64	14.29	0.58	14.17	0.71	0.715
Relative hand length	10.99^a^	0.55	10.68	0.41	10.65	0.52	0.049
Brachial index	74.47^a^^b^	2.95	77.01	3.12	76.00	3.34	0.041
Hand breadth/hand length	34.14	2.40	33.65	2.46	33.64	2.73	0.767
Relative biacromiale breadth	23.61	1.08	23.60	1.07	22.99	1.45	0.145
Relative biiliocristale breadth	16.30^a^	0.99	16.12^a^	1.04	15.56	0.84	0.028
FMpct	17.00	5.53	17.25	4.12	18.97	4.13	0.326
Endomorphy	2.17	0.67	2.32	0.80	2.34	0.79	0.734
Mesomorphy	6.00	1.62	5.87	1.08	5.35	1.10	0.190
Ectomorphy	1.99	1.03	1.92	0.77	2.47	1.03	0.107
Relative strength	3.01	0.65	2.86	0.63	2.80	0.85	0.909

^a^ significantly different from 3.

^b^ significantly different from 2.

**Fig 1:**
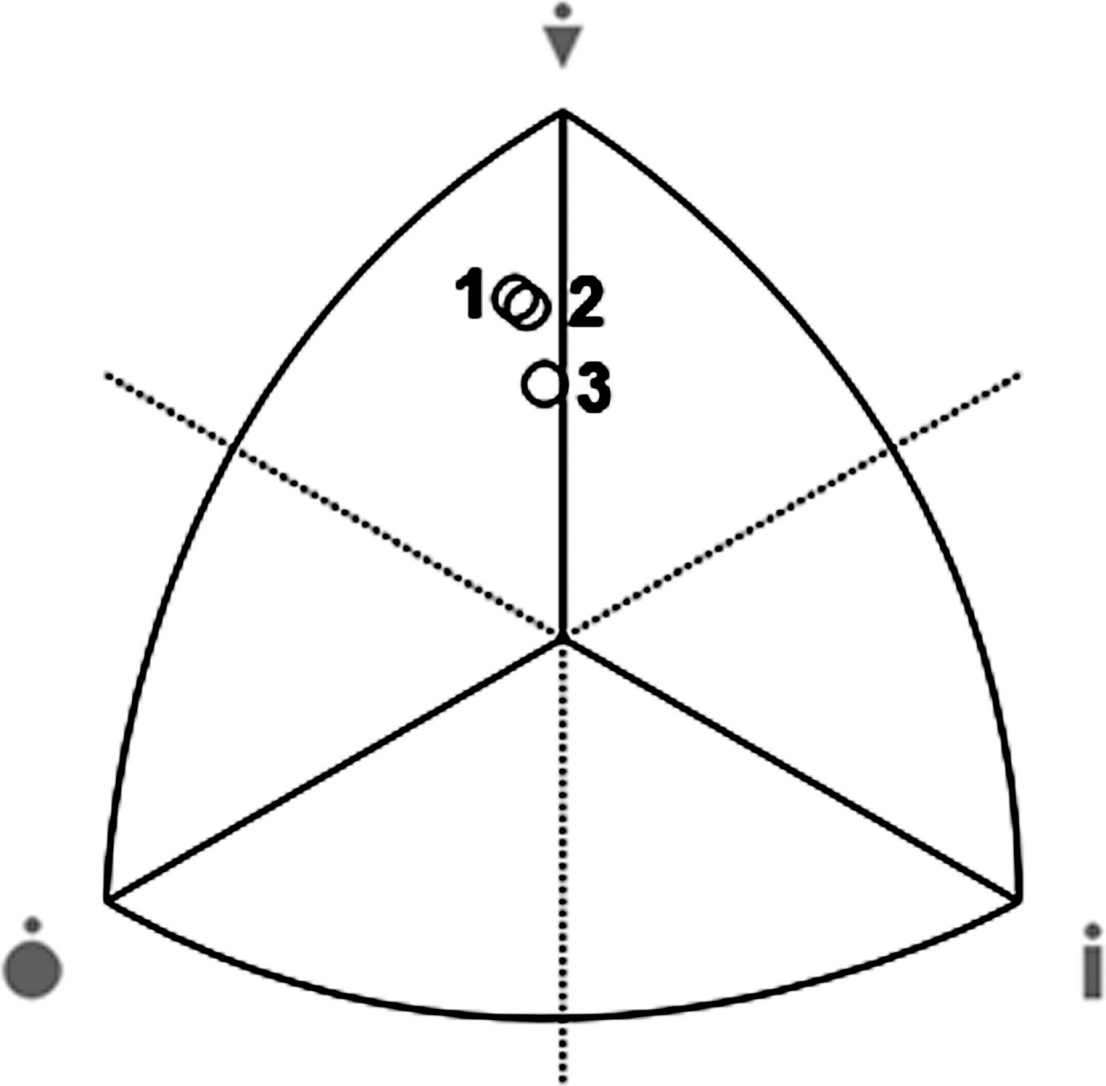
Distribution of somatotypes in groups of handball players (1 –super league, 2 – 1^st^ league, 3 –academic players).

Although the subjects in the third group have the highest percentage of fat in body weight, the differences do not reach the threshold of statistical significance. Similarly, there was no significant variation in the size of the body build components. Endomorphy reaches its highest values in groups 2 and 3. Mesomorphy is the highest among athletes in group 1, while ectomorphy is the highest in group 3. Subjects are characterized by a balanced mesomorph build (Fig 1), and somatotypes as a whole show no statistically significant intergroup variation (F = 1.75 p = 0.18). The differences in the magnitude of relative strength are also statistically insignificant. The highest values of the index characterize the players presenting the highest sports level, while the average values of the discussed characteristic in groups 2 and 3 are similar.

Anthropometric characteristics that are not modified by training were selected for discriminant analysis (Table 3). Only one canonical root was found to be statistically significant (χ^2^ = 36.03, p 0.000). All selected variables were included in the model (hand length, arm length, forearm length, arm span, body height, lower limb length). The cumulative proportion of explained variance corresponding to this function was approximately 88%. The structural coefficients indicate that the first discriminant function is weighted most heavily by the hand length, arm length, arm span, and lower limb length, and thus can be largely interpreted as characterizing the morphological conditions of throwing performance. The second function is defined by body height.

**Table 3:** Summary of stepwise discriminant analysis by team.

Step	Variable	Wilks’ lambda			F				Structure coefficients	
		Statistic	df1	df2	Statistic	df1	df2	*p*	Root 1	Root 2
1	Hand length	0.812	2	66	7.645	2	66	0.001	0.57	0.13
2	Arm length	0.753	2	65	4.941	4	130	0.001	0.69	0.33
3	Forearm length	0.692	2	64	4.307	6	128	0.001	-0.71	-0.54
4	Arm span	0.656	2	63	3.690	8	126	0.001	0.77	-0.75
5	Body height	0.615	2	62	3.413	10	124	0.001	-1.24	0.97
6	Lower limb height	0.567	2	61	3.336	12	122	0.000	0.74	-1.13

## Discussion

Our study presents the results of a detailed analysis of anthropometric characteristics and isometric strength in three groups of handball players representing different levels of sports competence. With the exception of subcutaneous fatness, we found that there were clear gradients in the magnitude of somatic features according to sportive level. The highest average values of body height and correlated characteristics of length (upper and lower limbs) and width (shoulder and hip width) were found in elite players while the lowest–among academic players. This confirms the opinions of other researchers that along with perfectly mastered agility-technical-tactical actions, better physical conditions can become an important and even decisive factor for effective playing [4]. We found no significant differences in subcutaneous fatness on the trunk. Slightly thicker subscapular and supraspinale skinfolds characterized the lowest ranked players. On the other hand, statistically significant differences were shown in skinfolds on the extremities. They were the thickest among academic athletes, while the differences between Super League and 1^th^ League players were negligible. This analysis confirms the results obtained by other authors who showed that high levels of physical activity and sports training are factors that influence the reduction of fatness. Nevertheless, not all regional fat deposits respond in the same way to exercise load [28, 29].

Although the results of the isometric strength and relative strength tests do not differ significantly between the groups of athletes, it should be noted that the mean values also decrease according to the gradient from the Super League team to the academic athletes. It is important to note that Super League players are the most homogeneous in terms of strength measurements, as evidenced by lower standard deviations than the other groups. The homogeneity of this group also distinguishes players at the international level from players at the national level. Our result was confirmed by the results obtained in tests assessing the explosive strength of lower limbs of handball players, such as squat jump and countermovement jump [30]. The results of previous studies suggest that motor skills, especially muscle strength (hand grip, trunk extensors and flexors) are important determinants of throwing velocity in handball [31, 32]. However, it should be mentioned that the isometric test used in this study is not the optimal method for evaluating a dynamic activity, such as throwing in handball and, as a result, may not accurately assess the potential performance of the muscles involved [33].

The goal of a handball game is to score as many goals as possible. Earlier studies have shown that the two most important determinants of effectiveness when throwing the ball into the opponent’s goal are speed and accuracy [34]. The speed of the throw is important for success, because the faster the ball travels to the goal, the less time defenders and the goalkeeper have to defend the shot. Throwing velocity is the resultant of factors such as technique, coordination of different body segments over time, and strength of upper and lower body muscle groups [35, 36]. During a throw, the muscles of the trunk are involved in the transfer of force from the lower body to the upper body, so a stronger and more stable lumbopelvic complex may contribute to greater rotational velocity in multi-segmental movements. The occurrence of significantly higher biiliocristal diameter in Super League players can be associated with this, which has also been confirmed in other studies [5, 37].

In addition to musculature, biomechanical conditions of movement execution also play an important role in the performance of motor tasks, and the specificity of the morphological structure need not be limited only to parameters characterizing the overall size [38]. Also, having unique body proportions can benefit an athlete in certain activities. The body proportions of adult athletes do not change under the influence of training, because the growth of bone dimensions in length is complete. In our study, after calculating indices that precipitate the effect of body height on somatic characteristics, we noticed that the highest ranked athletes were characterized by a significantly more massive physique, considered an advantageous characteristic in direct competition [16]. In addition, previous studies have shown that weight and height proportions are at significantly higher levels among elite athletes [39]. Furthermore, we demonstrated significantly greater upper limb span, longer lower and upper limbs, longer arm and hand in Super League players. This effect is not surprising given the results of analyses which indicate that throwing velocity in handball is strongly related to lower limb strength, jumping, and sprinting capabilities [40, 41]. There is an obvious positive effect of the great arm span and long upper limbs, which are helpful during the execution of the throw, as they condition a larger radius of action and are also beneficial in some defensive actions (e.g. block) [4]. The size of the hand has a significant effect on the throw in handball, with the larger dimensions allowing for better handling of the ball [36], and is considered a good predictor of throwing accuracy due to its positive correlation with maximum grip strength [42].

On the other hand, jumping capability assessed by vertical jump correlates with the position of the body’s center of gravity and lower limb dimensions. Research has shown that athletes with longer lower limbs perform better in vertical jump and their anaerobic power is higher [43, 44]. The higher jump height is due to the position of the center of mass, which shows a directly proportional relationship to lower limb length [45]. Unlike the previously discussed indices, the relative sitting height (cormic index) and the proportion between forearm and arm length (brachial index) were significantly lower among elite players. The ratio of sitting height to body height also provides information on the location of the center of mass. Although lower values of the mentioned index have a negative effect on agility and speed of movement, they have a positive effect on jumping [46]. Brachial index, on the other hand, expresses the ratio of the radius length to the humerus length and, as studies has shown, directly affects the mechanical advantage of the athlete’s upper limb in generating force, speed and power [47, 48], and shows high correlations with throwing velocity [49].

The body composition components of endomorphy, mesomorphy, and ectomorphy did not significantly differ between athlete groups. Mesomorphy was the highest among Super League subjects and the lowest among academic handball players, who had the highest endomorphy. Nikolaidis and Ingebrigtsen [40] observed similar trends manifested in the size of the structure components when analyzing the somatic structure of Greek handball players from teams differing in their position in the league ranking. However, it should be noted that endomorphy was at a lower level in our study. In contrast, mesomorphy among Greek handball players was slightly lower compared to our results. The level of endomorphy corresponds to the percentage of body fat and was the lowest among the highest-level athletes, as confirmed by the results obtained by other authors [40, 50]. The same somatotype–balanced mesomorph was present in all groups, which means that at all levels of play the subjects had a similar body structure.

We performed discriminant analysis to determine the set of variables that allow the best discrimination of groups of handball players presenting different sport levels. We selected features that do not change under training. All selected variables were included in the model; however, their interpretation requires great caution because the total number of cases is insufficient when six variables are included. Indeed, previous studies have showed that the coefficients of the discriminant function can be unstable when the number of cases is not twenty times the number of variables [51]. We showed that anthropometric variables affecting throwing performance (hand length, arm length, upper limb span, and lower limb length) weighed most heavily on the first discriminant function. The second function that explains only about 12% of the total discriminative power is determined by body height. This result is supported by previous studies that found a decreasing effect of body height on throwing speed with age [52].

## Conclusion

The results show that there is a relationship between different levels of play and morphological structure. Non-elite players compare unfavorably to Super League players in terms of numerous anthropometric characteristics and muscle strength. Handball players at the highest level dominate with their overall size and massive physique, which give them an advantage in direct contact on the field. All groups had the same somatotype (balanced mesomorph) which suggests that a similar physique characterizes players at all levels of play. However, elite players are characterized by specific body proportions that determine the biomechanical conditions conducive to optimizing the structure of movements relevant to handball. Stepwise discriminant analysis showed that throwing-related characteristics (hand length, arm length, upper limb span, lower limb length) accounted for 88% of the variance in team ranking, and can be used to identify morphological predispositions to play handball. In addition to that, the morphological approach may be one of the important factors leading to a good result in the complex talent selection process in sports.

## Supporting information

S1 Data (CSV)
